# Fluctuating or progressive hearing loss in the middle-to-high frequencies may suggest the neurotologic endotype of perilymphatic fistula without antecedent traumatic events

**DOI:** 10.3389/fneur.2025.1571379

**Published:** 2025-04-14

**Authors:** Han Matsuda, Yukihide Maeda, Tomoyasu Kitahara, Masafumi Sawada, Hiroe Kudo, Kei Sakamoto, Atsuya Takayama, Tetsuo Ikezono

**Affiliations:** Department of Otorhinolaryngology, Saitama Medical University, Saitama, Japan

**Keywords:** Perilymphatic fistula, antecedent traumatic events, idiopathic sudden sensorineural hearing loss, Cochlin-tomoprotein, fluctuating hearing loss, progressive hearing loss, high-frequency hearing loss, fistula sign

## Abstract

**Background:**

Idiopathic sudden sensorineural hearing loss (ISSNHL) is a common clinical condition. Recent studies indicate that approximately 20% of ISSNHL cases may involve perilymphatic fistula (PLF). The detection of Cochlin-tomoprotein (CTP) in middle-ear lavage samples confirms the diagnosis of PLF.

**Aims/Objectives:**

To clarify the clinical characteristics of inner ear–related symptoms in patients with PLF who lacked any antecedent traumatic events prior to symptom onset.

**Materials and methods:**

We retrospectively reviewed clinical records and CTP test results in 769 cases from 70 hospitals in Japan.

**Results:**

Among these cases, 204 had no history of antecedent events. CTP-positive findings were more frequently observed in patients exhibiting fluctuating and/or progressive hearing loss than in those without these symptoms (*p* < 0.05, Fisher’s exact test). The odds of a positive CTP test did not differ between patients with and without vestibular symptoms, nystagmus, a fistula sign, a popping sensation, or streaming water–like tinnitus (*p* > 0.05, Fisher’s exact test). The CTP positivity rate was highest in patients with a high-frequency sloping audiogram.

**Conclusions and significance:**

Fluctuating or progressive hearing loss in the middle-to-high frequencies may reasonably suggest PLF in the absence of antecedent traumatic events.

## 1 Introduction

Perilymphatic fistula (PLF) is defined as an abnormal communication between the perilymph-filled inner ear and the air-filled middle ear or mastoid, or cranial spaces. PLF causes hearing loss and vestibular symptoms, and high cure rates following PLF repair have been reported in multiple studies (1–3). Because PLF arises from a distinct pathological mechanism that can often be corrected with appropriate surgical treatment, it may be viewed as an “endotype” of neurotologic diseases. Historically, few data have been published on the incidence or prevalence of PLF because a definitive diagnosis depended on the invasive procedure of exploratory tympanotomy. However, Cochlin-tomoprotein (CTP) has recently emerged as a reliable protein biomarker for PLF. CTP, which is specifically detected in perilymph fluid, can be identified by enzyme-linked immunosorbent assay (ELISA) in lavage fluid from the middle ear cavity, enabling preoperative PLF diagnosis (4). In Japan, this laboratory test has been covered by public health insurance since 2022.

Idiopathic sudden sensorineural hearing loss (ISSNHL) is a common otologic condition marked by sudden onset of hearing loss without a known cause; its annual incidence is approximately 60.9 per 100,000 individuals in Japan (5). Unfortunately, no treatment has yet shown definitive efficacy in randomized controlled trials, and about two-thirds of patients develop lifelong hearing deficits that negatively affect their quality of life (6). A recent Japanese study revealed that 22% of patients who met diagnostic criteria for ISSNHL actually had PLF based on a positive CTP test (7). This finding is especially significant because PLF can often be successfully managed with surgical repair.

Clinical histories in PLF can be classified into four categories based on the nature of potential antecedent events: (1) trauma and middle/inner ear diseases (e.g., cholesteatoma, congenital anomalies, stapes surgery, cochlear implantation); (2) barotrauma resulting from external factors (e.g., diving, air travel, blast explosions); (3) barotrauma caused by internal pressure changes (e.g., nose blowing, sneezing, lifting); and (4) no discernible antecedent event. In an earlier study by Matsuda et al., clinical symptoms related to the inner ear were examined in 497 otologic patients, including those who tested positive for CTP as well as those who tested negative (8). Approximately half of the 497 patients reported no antecedent event. Without CTP testing, these “category 4” cases would often be diagnosed as ISSNHL by conventional diagnostic criteria. In this paper, we describe the clinical presentations and audiometric findings of CTP-positive, category 4 patients. Our aim is to provide practical guidance for the preoperative diagnosis of PLF in cases with no identifiable precipitating trauma.

## 2 Materials and methods

### 2.1 Study subjects

This retrospective study reviewed clinical histories and the results of Cochlin-tomoprotein (CTP) detection tests for 769 cases from 70 hospitals in Japan between April 2014 and August 2016. Patients were suspected to have PLF based on their clinical histories (trauma or middle/inner ear diseases) and/or inner ear–related manifestations. Some of these data were previously reported (8), but the present study includes additional analyses and novel findings. A list of participating hospitals is provided in the Acknowledgments. We have selected idiopathic cases in this study (Table 1) (3, 9). As defined in Table 1 created by the Japanese PLF study group, idiopathic cases correspond to Category 4, excluding Category 1 cases with a history of previous otological surgery, ear diseases, or direct trauma. Accordingly, temporal bone fractures, inner ear anomalies, and dehiscence were excluded based on high-resolution computed tomography (HRCT) findings. Similarly, Category 2 and 3 cases who had experienced internal or external barotraumatic events were also excluded.

**Table 1:** Categorization of PLF cases formulated by a Japanese study group.

▪Category 1 Linked to trauma, middle, and/or inner ear diseases, surgeries (1) a Direct labyrinthine trauma (stapes luxation, otic capsule fracture, etc.) b Other trauma (head injury, body contusion, etc.) (2) a Middle or inner ear diseases (cholesteatoma, tumor, anomaly, dehiscence, etc.) b Iatrogenic (ear surgeries, medical treatments, etc.)
▪Category 2 Linked to barotrauma caused by antecedent events of external origin (such as flying or diving)
▪Category 3 Linked to barotrauma caused by antecedent events of internal origin (such as straining, sneezing, or coughing)
▪Category 4 Has no apparent antecedent event (idiopathic) “Spontaneous” should not be used.

Protocols for collecting middle-ear lavage samples were approved by the local ethics committee of each institution, and written informed consent was obtained from all subjects. Approval by the Institutional Review Board (IRB) of Saitama Medical University was granted under protocol number 13–086 (Principal Investigator: T.I.). All procedures were performed in accordance with the Declaration of Helsinki.

### 2.2 CTP detection test

In all patients, CTP concentrations were measured via enzyme-linked immunosorbent assay (ELISA) in lavage fluid obtained from the middle-ear cavity. The assay was conducted by SRL, Inc. (Tokyo, Japan) using an ELISA kit with a specific polyclonal antibody. Based on established criteria (4), the diagnostic cut-off values for CTP were as follows:

- **CTP < 0.4 ng/mL:** Negative
- **0.4 ≤ CTP < 0.8 ng/mL:** Intermediate
- **CTP ≥ 0.8 ng/mL:** Positive

In this study, cases with CTP ≥ 0.8 ng/mL were classified as CTP-positive, whereas those with CTP < 0.8 ng/mL were categorized as CTP-intermediate or CTP-negative.

### 2.3 Data collection

From the initial 769 cases, we selected those who had no known antecedent traumatic events prior to onset of inner ear–related symptoms (Category 4). Among these cases, patients presenting with hearing loss who underwent CTP testing within 30 days of symptom onset were further identified for analysis.

The following data were extracted from patient charts: (1) CTP test result; (2) patient age; (3) baseline hearing level (before any treatment; hereafter referred to as “hearing level”); and (4) time interval (days) from symptom onset to the CTP test (hereafter referred to as “duration”). We also noted the presence or absence of six clinical features: (1) fluctuation and/or progression of hearing loss (defined as ≥10 dB change), (2) vestibular symptom, (3) nystagmus, (4) fistula sign, (5) popping sensation, and (6) streaming water–like tinnitus.

Audiogram configurations were classified according to modified criteria by Demeester et al. (10), including flat, high-frequency sloping, low-frequency ascending, mid-frequency “U,” mid-frequency reversed “U,” and deafness/scale-out types. The audiogram configuration was determined based on the audiogram recorded at the time of the most severe hearing loss.

### 2.4 Statistical analyses

Patient age, hearing level, and duration (days) were compared between the CTP-positive group and the CTP-intermediate/negative group using the Mann–Whitney *U* test. The odds of CTP positivity were compared between patients with and without each of the six clinical features using Fisher’s exact test. All statistical analyses were performed using JMP Pro 16 (SAS Institute, Cary, NC), and statistical significance was defined as *p* < 0.05.

## 3 Results

**Figure 1:**
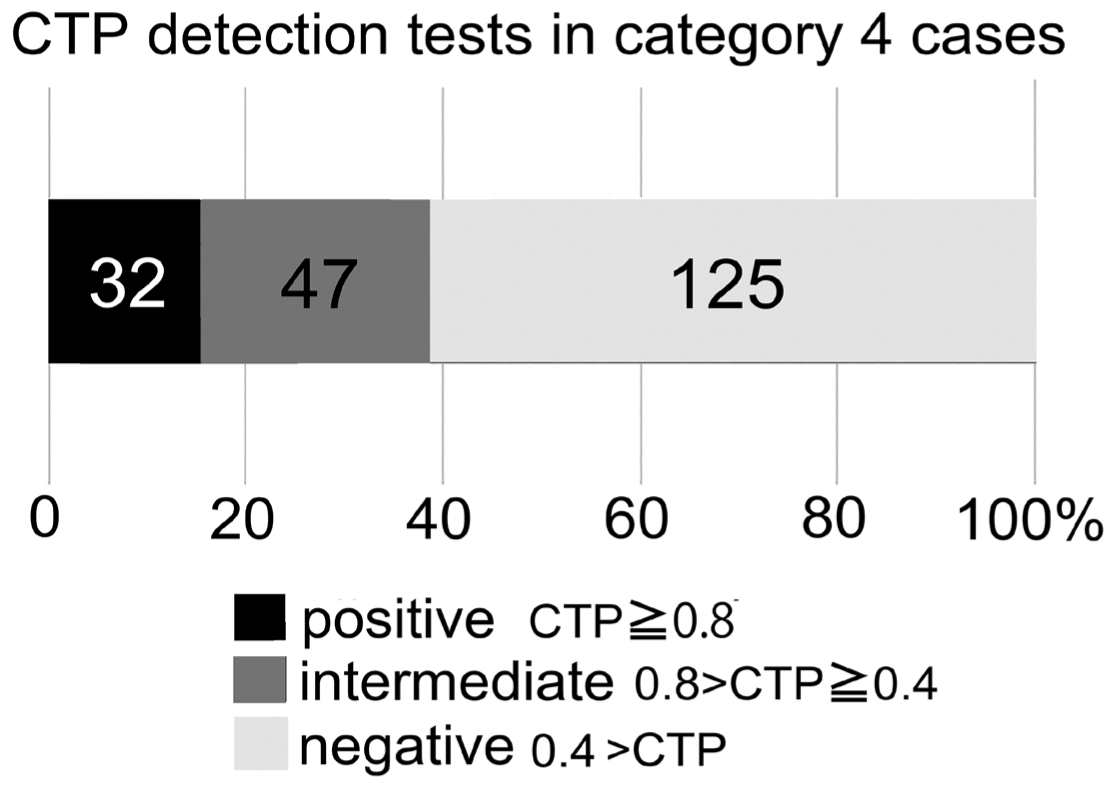
Results of CTP detection tests in patients without antecedent traumatic events (Category 4 cases). Among 204 Category 4 cases, 32 (15.7%) tested positive and 172 (84.3%) were intermediate or negative.

Of the 769 total cases, 381 cases were idiopathic (Category 4). Among these 381 cases, 206 underwent CTP testing within 30 days of symptom onset, and 2 were excluded because they did not exhibit hearing loss. Consequently, 204 patients were included in the final analysis. Of these, 125 had CTP < 0.4 ng/mL, 47 had 0.4 ≤ CTP < 0.8 ng/mL, and 32 had CTP ≥ 0.8 ng/mL. Thus, 32 patients (15.7%) were classified as CTP-positive, and the remaining 172 (84.3%) were classified as CTP-intermediate or negative (Figure 1).

The mean (± SD) patient age was 57.7 ± 17.5 years, mean hearing level was 81.4 ± 23.8 dB HL, and mean duration from symptom onset to CTP testing was 9.2 ± 6.2 days. None of these parameters differed significantly between the CTP-positive and CTP-intermediate/negative groups.

**Figure 2:**
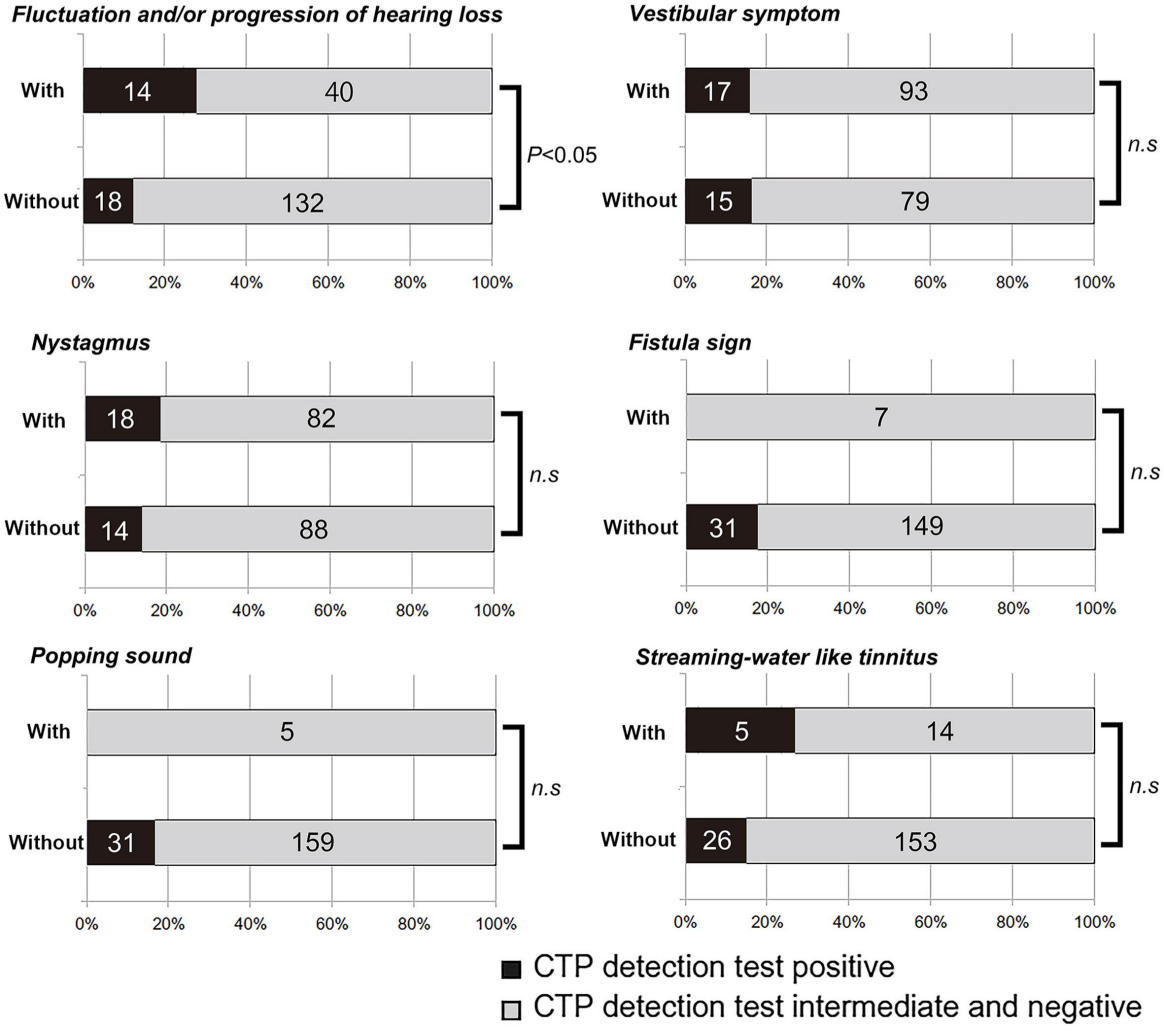
Results of CTP detection tests in patients with or without six clinical characteristics suggesting perilymphatic fistula (PLF). Positive CTP results occurred more frequently in patients with fluctuating or progressive hearing loss (*p* < 0.05, Fisher’s exact test). No significant difference was observed for vestibular symptoms, nystagmus, fistula sign, popping sensation, or streaming water–like tinnitus.

CTP positivity was more frequently observed in patients with fluctuating and/or progressive hearing loss (14/54, 25.9%) than in those without such changes (18/150, 12%, *p* < 0.05, Fisher’s exact test). However, the odds of CTP positivity did not differ significantly between patients with and without vestibular symptoms, nystagmus, a fistula sign, popping sound, or streaming water–like tinnitus (Figure 2).

Among the CTP-positive cases, flat-types audiograms (15 cases) were most frequently observed, followed by high-frequency sloping (5 cases), a deafness/scale-out pattern (5 cases), a mid-frequency “U” (1 case), whereas low-frequency ascending and mid-frequency reversed “U” types were absent.

Among the various audiogram configurations, CTP positivity was observed in 15/104 (14.4%) patients with a flat audiogram, 5/20 (25%) with high-frequency sloping, 0/6 (0%) with low-frequency ascending, 1/7 (14.3%) with a mid-frequency “U,” 0/2 (0%) with a mid-frequency reversed “U,” and 5/52 (9.6%) with a deafness/scale-out pattern (Table 2).

**Table 2:** CTP detection test results by audiogram configuration.

Configuration of audiogram	CTP positive	CTP intermediate and negative	% of CTP positive
Flat	15	89	14.4
High-frequency sloping	5	15	25
Low-frequency ascending	0	6	0
Mid-frequency U	1	6	14.3
Mid-frequency reversed U	0	2	0
Deafness and scale-out type	5	47	9.6

High-frequency sloping audiograms were associated with the highest rate of positive CTP findings. None of the patients with low-frequency ascending audiograms were CTP-positive.

## 4 Discussion

In patients suspected of having PLF, a positive Cochlin-tomoprotein (CTP) detection test confirms the diagnosis in accordance with the diagnostic criteria set by the Japanese Ministry of Health, Labour, and Welfare (8). These criteria also acknowledge idiopathic PLF in some patients without prior traumatic events. Therefore, in cases of idiopathic sudden sensorineural hearing loss (ISSNHL), it is reasonable to perform a CTP detection test to differentiate PLF. If clinical findings strongly suggest PLF, PLF repair surgery may be warranted.

Our data indicate that among the Category 4 patients (those without antecedent traumatic events or other ear related medical history), those exhibiting fluctuating or progressive hearing loss are more likely to have PLF than those who do not. Notably, fluctuating or progressive hearing loss is also characteristic of hydropic ear disease (HED), which is recognized as an endotype of clinical Ménière’s disease (11). Consequently, differentiating PLF from HED can be challenging in patients who present with fluctuating hearing loss and vestibular symptoms in the absence of trauma. Recent advances in imaging techniques allow for visualization of endolymphatic hydrops (ELH) via 3-Tesla MRI after intravenous gadolinium injection (12). However, cochlear ELH has been reported in up to 10% of asymptomatic individuals with no hearing loss or vestibular symptoms (13), suggesting that ELH is not necessarily pathognomonic. Moreover, experimental studies have shown that PLF can induce ELH in the temporal bone of animal models (14), indicating that ELH on imaging does not exclude a possible PLF.

In our cohort, the CTP positivity rate was highest in patients with high-frequency sloping audiograms (25%), followed by those with flat (14.4%), mid-frequency U (14.3%), and deafness/scale-out (9.6%) configurations; notably, no patients with low-frequency ascending audiograms tested positive for CTP. These results suggest that PLF may preferentially involve middle-to-high frequency hearing loss, whereas HED generally affects the low frequencies. Thus, fluctuating or progressive hearing loss in the middle-to-high frequencies may be associated with PLF rather than HED, and it is further distinguishable from age-related high-frequency hearing loss by the presence of fluctuation, which characterizes PLF.

Other classic clinical findings traditionally associated with PLF—such as vestibular symptoms, nystagmus, fistula sign, popping sensation, and streaming water–like tinnitus (9, 15, 16)—were not significantly correlated with positive CTP results in our dataset. This lack of statistical significance may be due to the small number of patients exhibiting a fistula sign (*n* = 7) or a popping sensation (*n* = 5). Consequently, larger sample sizes may be required to confirm any association between these clinical features and a positive CTP test.

It is important to have a clear clinical question: Is the condition referred to as PLF in this paper the same as or different from dehiscence syndrome? The CTP test detects PL leakage from a small fistula, which is typically undetectable by HRCT, and this leakage can lead to fluctuating or progressive hearing loss, sudden deafness, or vestibular symptoms. This condition differs from third mobile window syndrome, such as Superior Canal Dehiscence Syndrome (SCDS), which is typically characterized by autophony, bone conduction hyperacusis, phonophobia, Tullio phenomenon, and oscillopsia. A key diagnostic finding for SCDS is hyperreactive VEMP; however, this has never been observed in our clinical practice in PLF cases. VEMP is routinely performed in our clinic when indicated. In a previously published retrospective study, 22 patients underwent PLF repair surgery (3), and none were found to have dehiscence. Regarding VEMP testing, of the 12 patients from these 22 cases who underwent cervical and ocular VEMP, seven exhibited reduced responses in the affected ear, while five showed normal responses comparable to the contralateral normal ear. Thus, based on symptoms, CT findings, and VEMP results, PLF and SCDS can be considered distinct conditions.

Otic capsule dehiscence syndrome (OCDS) has been reported even in cases with normal CT findings. Among the eight patients with “otic capsule defects not visualized with imaging” reported by Wackym et al. (17), six exhibited decreased cVEMP thresholds and increased amplitudes. In contrast, in our routine clinical practice, we have performed cVEMP and oVEMP in patients with PLF, yet we have not observed such characteristic VEMP findings in any cases. Based on these observations, we consider the PLF cases classified as Category 4 in our study to be distinct from the condition described by Wackym et al. (17) as “otic capsule defects not visualized with imaging.”

Overall, our findings suggest that fluctuating or progressive hearing loss in the middle-to-high frequencies may reasonably indicate PLF even in the absence of any precipitating traumatic event. In such cases, we recommend confirming the diagnosis by CTP detection testing and, if positive, proceeding with PLF repair surgery.

### 4.1 Limitation of the study

In the patient cohort of this multicenter study, VEMP was not performed in all patients. Consequently, the dataset lacks information on VEMP which may be important for the diagnosis of PLF to distinguish from OCDS.

## Data Availability

The raw data supporting the conclusions of this article will be made available by the authors, without undue reservation.
